# Prelabour Rupture of Membranes among Pregnant Women Visiting a Tertiary Care Centre: A Descriptive Cross-sectional Study

**DOI:** 10.31729/jnma.8186

**Published:** 2023-06-30

**Authors:** Yam Prasad Dwa, Sunita Bhandari, Manisha Bajracharya

**Affiliations:** 1 Department of Obstetrics and Gynecology, KIST Medical College and Teaching Hospital, Gwarko, Lalitpur, Nepal

**Keywords:** *miscarriage*, *oligohydramnios*, *prevalence*

## Abstract

**Introduction:**

Prelabour rupture of membranes is a common obstetrics problem associated with maternal and perinatal morbidity and mortality. The exact cause is not known although various factors are found to be related to this condition. Hence, the objective of this study was to find out the prevalence of prelabour rupture of membranes among pregnant women in a tertiary care centre.

**Methods:**

This was a descriptive cross-sectional study conducted from 1 November 2021 to 30 November 2022. Ethical approval was taken from the Institutional Review Committee (Reference number: 2078/79/49). A structured proforma was filled out after taking a detailed history from each pregnant woman. Convenience sampling method was used. Point estimate and 99% Confidence Interval were calculated.

**Results:**

Among 700 pregnant women, the prevalence of prelabour rupture of membranes was 56 (8%) (5.36-10.64, 99% Confidence Interval). Among them, 40 (71.43%) occurred in the term, while preterm (before 37 weeks) occurred in 16 (28.57%) of all pregnancies. Previous miscarriage occurred in 15 (26.78%) followed by gestational diabetes mellitus 8 (14.28%).

**Conclusions:**

The prevalence of prelabour rupture of membranes was found to be lower than other studies done in similar settings.

## INTRODUCTION

Prelabour rupture of membrane (PROM) is the spontaneous rupture of the membrane before the onset of labour.^1^ In most cases, this occurs near term, but when membrane rupture occurs before 37 weeks gestation, it is known as the preterm prelabour rupture of membranes (PPROM). PROM complicates approximately 8% of pregnancies in the term,^1^ while PPROM (before 37 weeks) occurs in approximately 3% of all pregnancies and accounts for one-third of preterm births.^2^ PROM is a common obstetrics problem associated with maternal and perinatal morbidity and mortality.^3^

The exact cause of PROM is not known and the causes could be multifactorial. There are various factors that are related to PROM including prior preterm birth, cigarette smoking, polyhydramnios, urinary and sexually transmitted infection, prior PROM, work during pregnancy, low body mass index, bleeding, and low socioeconomic status.^4,5^

The aim of this study was to find out the prevalence of prelabour rupture of membranes among pregnant women in a tertiary care centre.

## METHODS

This was a hospital-based descriptive cross-sectional study conducted from 1 November 2021 to 30 November 2022 after obtaining ethical approval from the Institutional Review Committee (Reference number: 2078/79/49) from the Department of Obstetrics and Gynecology at KIST Medical College and Teaching Hospital, Gwarko, Lalitpur, Nepal. Patients were informed regarding the study and informed written consent was taken prior to the study. All the admitted pregnant women from the gestational age of 28 weeks and above were recruited. Pregnant women with onset of labour within <1 hour of rupture of membrane or with artificial rupture of membranes were excluded. Convenience sampling method was used. The sample size was calculated by using the following formula:


$$\begin{array}{ll} \text{n} & ={\text{Z}}^{2}\times \frac{\text{p}\times \text{q}}{{\text{e}}^{\text{2}}}\\ & =2.57^{2}\times \frac{0.50\times 0.50}{0.05^{2}}\\ & =661 \end{array}$$

Where,

n = minimum required sample size
Z = 2.57 at 99% Confidence Interval (CI)
p = prevalence taken as 50% for maximum sample size calculation
q = 1-p
e = margin of error, 5%

The minimum calculated sample size was 661. However, the final sample size taken was 700.

The diagnosis of PROM was based on a maternal history of the passage of a gush of fluid per vagina before the onset of labour plus visualization of pooling of amniotic fluid in the posterior vaginal fornix or/and direct visualization of fluid leakage from the cervical canal per speculum examination. Ultrasonographic demonstration of oligohydramnios was taken as evidence of PROM where the history or clinical examination is inconclusive. Thorough history regarding sociodemographic and obstetrics variables was obtained from the patients, and risk factors such as previous miscarriage, previous preterm delivery, previous PROM, cervical surgery/ cerclage, previous cesarean section, abnormal vaginal discharge /genital infection in pregnancy, urinary tract infection, abdominal trauma, coitus in the third trimester, cigarette smoking during pregnancy, bleeding during pregnancy, polyhydramnios, chronic hypertension/ pregnancy-induced hypertension (PIH), diabetes/ gestational diabetes mellitus (GDM), thyroid disorder and anaemia during index pregnancy were documented in structured proforma.

Data were entered in Microsoft Excel and analyzed using IBM Statistics SPSS 18.0. Point estimate and 99% CI were calculated.

## RESULTS

Among 700 pregnant women, the prevalence of prelabour rupture of membranes was 56 (8%) (5.3610.64, 99% CI).

Out of 56 patients, 25 (44.64%) were in the age group 25-29 years. Twenty-one (37.50%) women were involved in physical efforts and standing work. Fifty-one (91.07%) belong to middle-class families (Table 1).

**Table 1:** Sociodemographic variable (n = 56).

Variables	Category	n (%)
Age (in years)	<19	2 (3.57)
	20-24	14 (25.00)
	25-29	25 (44.64)
	30-34	10 (17.85)
	>35	5 (8.92)
Type of work	Physical efforts	21 (37.50)
	Standing	21 (37.50)
	Sitting	14 (25.00)
	lifting heavy objects	-
Socioeconomic status	Low	1 (1.78)
	Middle	51 (91.07)
	High	4 (7.14)

There were equal number of primi and multi gravida 28 (50%) with nulli parity in 30 (53.57%) cases and most of them were booked cases 48 (85.71%) with ≥ 4 ANC visits 52 (92.85%). Twin pregnancy was seen in 2 (3.57%) cases and 4 (7.14%) had malpresentation in the index pregnancy (Table 2).

**Table 2:** Obstetrical variables (n = 56).

Variables	Category	n (%)
Gestational age (weeks)	28-36 weeks+6 days	16 (28.57)
	37-40	39 (69.64)
	40-41	1 (1.78)
Gravida	Primi	28 (50)
	Multi	28 (50)
Parity	0	30 (53.57)
	1	20 (35.71)
	2	4 (7.14)
	3	2 (3.57)
	≥4	0 (0)
Previous	Spontaneous	8 (14.28)
miscarriage	Induced	7 (12.50)
Mode of termination of previous miscarriage	Medical abortion (MA)	3 (5.35)
	Manual vaccuum aspiration (MVA)	3 (5.35)
	Medical induction (MI)	1 (1.78)
	Dilatation and evacuation (D and E)	2 (3.57)
Previous route of delivery of last child	Normal delivery	18 (32.14)
	Instrumental delivery	1 (1.78)
	Cesarean section	7 (12.50)
Antenatal care	Booked	48 (85.71)
	Unbooked	8 (14.28)
ANC visits	<4	4 (7.14)
	≥4	52 (92.85)
Type of index pregnancy	Single	54 (96.42)
	Twin	2 (3.57)
Presentation	Cephalic	52 (92.85)
	Breech	2 (3.57)
	Others	2 (3.57)

Among 56 women, 15 (26.78%) had a previous miscarriage, followed by diabetes in 8 (14.28%) women (Table 3).

**Table 3:** Causes related to PROM (n= 56).

Variables	n (%)
Previous miscarriage	15 (26.78)
Diabetes/ GDM during pregnancy	8 (14.28)
History of previous cesarean section	7 (12.50)
Sexual intercourse in 3rd trimester	6 (10.71)
History of previous preterm delivery	4 (7.14)
Urinary tract infection	4 (7.14)
Bleeding during index pregnancy	4 (7.14)
History of previous PROM	4 (7.14)
Abnormal vaginal discharge/genital infection in pregnancy	2 (3.57)
Chronic hypertension/PIH	2 (3.57)
Thyroid disorder during index pregnancy	2 (3.57)
Polyhydramnios	1 (1.78)
Anaemia during index pregnancy	1 (1.78)

## DISCUSSION

The prevalence of prelabour rupture of membranes was 56 (8%) among 700 pregnant women. Out of which 40 (71.42%) occurred in the term, while PPROM occurred in 16 (28.57%) of all pregnancies. This finding is similar to the previous study done in Nepal, which reported the prevalence of PROM to be 8.9%.^6^ This is also similar to a study done in Bangladesh with the prevalence of PROM of 8.2%.^7^ Term PROM was higher 39 (69.2%) than preterm PROM 17 (30.8%). Similarly, a study done in India reported prevalence as 9.8%.^8^ This study is also in agreement with the systematic review and meta-analysis,^9^ that showed the pooled prevalence of PROM was 9.2% among pregnant women in Ethiopia. In contrast, a multi-centric study in China,^10^ showed a high prevalence of PROM to be 18.72%. Also, a study done in Indonesia,^11^ found that the prevalence of PROM was 22.6%. The differences in prevalence could be due to the difference in the population studied.

Our study revealed the most commonly reported causes were previous miscarriage 15 (26.78%), diabetes/gestational diabetes mellitus 8 (14.28%), history of previous cesarean section 7 (12.50%), sexual intercourse in third trimester 6 (10.71%), previous preterm delivery, previous PROM, urinary tract infection, bleeding during pregnancy 4 (7.14%), abnormal vaginal discharge/genital infection in pregnancy, chronic hypertension/PIH, thyroid disorder 2 (3.57%), anaemia, and polyhydramnios during index pregnancy 1 (1.78%). This finding was similar to a previous study done in Nepal, where the frequently associated maternal risk factors were a history of prior abortion 19.5%, urinary tract infection 8.5%, and antecedent coitus 8.5%.^6^ In the study conducted in Iran, the maternal risk factors included diabetes 12.7%, hypertension 9.5%, smoking 8.9%, history of premature rupture of membrane 8.9%, urinary tract infection 7.2%, thyroid disorders 5%, history of preterm delivery 4.4%, and cerclage 3.8%.^12^ However, none of the women in our study had cigarette smoking and cervical cerclage/history of cervical surgery.

In the study conducted in Bangladesh, the study of factors revealed aetiology was unknown in 46 (6.8%), low socioeconomic condition 60.6%, anaemia 45%, lower genital tract infection 35.6%, UTI 31%, previous history of PROM 27.9%, malpresentation 15%, multiple pregnancies 6.7%, polyhydramnios 6%, history of recent coitus 12%, DM and GDM 10.5% were commonly associated with PROM.^7^ In consistency, our study also revealed twin pregnancy 2 (6.7%) and malpresentation 4 (7.14%) in the index pregnancy.

A study of factors of PROM in India showed the cause of most PROM cases was unclear but associated with the history of PROM. Most cases of PROM occurred in housewives aged 20 to 30 years old.^8^ Similarly, the maximum cases belonged to the age group 25-29 years 25 (44.64%). A study conducted in Nigeria showed the previous history of PROM, history of preterm delivery, low socioeconomic status, and genitourinary infection were highly predictive of PROM.^13^ In contrast, the majority of PROM cases were of middle socioeconomic status 51 (91.07%) in this study.

In a hospital-based study in Southern Ethiopia, previous history of abortion, lack of ANC, previous history of PROM, caesarean delivery, use of a maternal waiting room (MWR), and mid-upper arm circumference (MAUC) <23 cm were identified as determinants of PROM.^14^ However, the majority of cases had regular ANC with ≥4 ANC visits 52 (92.85%) in this study. In addition, another study in Southern Ethiopia (2022)^15^ showed hypertension during the index pregnancy (38.7%), history of abortion (37.3%), history of PROM (48%), and history of cesarean section (33.3%) were related to premature rupture of membrane.

There are a few limitations in this study. Since this is a descriptive study, the analytical parameters could not be evaluated. Also, the study is based on a single centre hence, the findings of this study cannot be generalized to the general populations across the nation.

## CONCLUSIONS

The prevalence of prelabour rupture of membranes was found to be lower than other studies done in similar settings. Timely identification of the causative factors can help clinicians in identifying at-risk women for intensified obstetric and neonatal care and also in formulating prevention programs.
